# Estimating average alcohol consumption in the population using multiple sources: the case of Spain

**DOI:** 10.1186/s12963-016-0090-4

**Published:** 2016-06-02

**Authors:** Luis Sordo, Gregorio Barrio, María J. Bravo, Joan R. Villalbí, Albert Espelt, Montserrat Neira, Enrique Regidor

**Affiliations:** National Epidemiology Center, Carlos III Health Institute, Avenida Monforte de Lemos 5, E-28029 Madrid, Spain; Consortium for Biomedical Research in Epidemiology & Public Health (CIBERESP), Avenida Monforte de Lemos 5, E-28029 Madrid, Spain; Department of Preventive Medicine and Public Health, Madrid Complutense University, Ciudad Universitaria s/n, E-28040 Madrid, Spain; National School of Public Health, Carlos III Health Institute, Avenida Monforte de Lemos 5, E-28029 Madrid, Spain; Agència de Salut Pública de Barcelona, Pl. Lesseps, 1. E-08023 Barcelona, Spain; Ministry of Health, Social Services and Equality, Paseo del Prado 18-20, E-28071 Madrid, Spain

**Keywords:** Alcohol, Data sources, Availability, Sales, Purchases, Consumption, Self-report, Population surveys, Underestimation

## Abstract

**Background:**

National estimates on per capita alcohol consumption are provided regularly by various sources and may have validity problems, so corrections are needed for monitoring and assessment purposes. Our objectives were to compare different alcohol availability estimates for Spain, to build the best estimate (actual consumption), characterize its time trend during 2001–2011, and quantify the extent to which other estimates (coverage) approximated actual consumption.

**Methods:**

Estimates were: alcohol availability from the Spanish Tax Agency (Tax Agency availability), World Health Organization (WHO availability) and other international agencies, self-reported purchases from the Spanish Food Consumption Panel, and self-reported consumption from population surveys. Analyses included calculating: between-agency discrepancy in availability, multisource availability (correcting Tax Agency availability by underestimation of wine and cider), actual consumption (adjusting multisource availability by unrecorded alcohol consumption/purchases and alcohol losses), and coverage of selected estimates. Sensitivity analyses were undertaken. Time trends were characterized by joinpoint regression.

**Results:**

Between-agency discrepancy in alcohol availability remained high in 2011, mainly because of wine and spirits, although some decrease was observed during the study period.

The actual consumption was 9.5 l of pure alcohol/person-year in 2011, decreasing 2.3 % annually, mainly due to wine and spirits. 2011 coverage of WHO availability, Tax Agency availability, self-reported purchases, and self-reported consumption was 99.5, 99.5, 66.3, and 28.0 %, respectively, generally with downward trends (last three estimates, especially self-reported consumption). The multisource availability overestimated actual consumption by 12.3 %, mainly due to tourism imbalance.

**Conclusions:**

Spanish estimates of per capita alcohol consumption show considerable weaknesses. Using uncorrected estimates, especially self-reported consumption, for monitoring or other purposes is misleading. To obtain conservative estimates of alcohol-attributable disease burden or heavy drinking prevalence, self-reported consumption should be shifted upwards by more than 85 % (91 % in 2011) of Tax Agency or WHO availability figures. The weaknesses identified can probably also be found worldwide, thus much empirical work remains to be done to improve estimates of per capita alcohol consumption.

**Electronic supplementary material:**

The online version of this article (doi:10.1186/s12963-016-0090-4) contains supplementary material, which is available to authorized users.

## Background

Per capita or average population alcohol consumption is a key indicator of alcohol exposure which reflects future volume and trends in alcohol-related problems and is essential to calculate the burden of alcohol-attributable disease. Thus, valid and comparable per capita consumption estimates are required to formulate and assess alcohol-related policies [1–6]. Estimates are usually selected from various sources at different stages of the distribution process from the time the beverages are available on the market for human consumption and actual consumption by the resident population. Consequently, important threats to their validity and comparability exist. For example, recorded alcohol sales based on excise duties on alcoholic beverages from national sources are generally regarded as the most valid proxy of per capita alcohol consumption in Western countries [1, 7–11]. However, in some countries like Spain, wine is exempt from excise duty. Moreover, this proxy mainly includes legal wholesale alcohol sales, which occur at the beginning of the marketing process and therefore do not adequately reflect alcohol consumption by residents. Thus, it must be corrected or adjusted to account for untaxed beverages, unrecorded alcohol sales, alcohol losses, and the balance of consumption/purchases by international visitors. Correction algorithms generally are not internationally standardized, are sometimes not even explicit, and are based on many parameters that require empirical data, which are often lacking and must be assumed or extrapolated from elsewhere [1, 6, 12–15]. Despite these threats to validity, the process by which individual countries make these estimates has rarely been assessed, nor have estimates from various sources been systematically compared.

Population surveys generally provide the prevalence of self-reported consumption of specified amounts of alcohol by different variables, which can be used directly for monitoring purposes and intervention assessment, or indirectly to estimate the burden of alcohol-attributable disease. However, surveys often largely underestimate per capita consumption derived from alcohol sales, often covering only 30–65 % of actual consumption [1, 7, 9, 14, 16–19], with spatiotemporal variations in coverage even when the same survey is used [6]. Thus, for some purposes, such as estimating the alcohol-attributable disease burden, self-reported consumption figures need to be shifted upwards. The elevation factor could be extrapolated from elsewhere, but given the between-country heterogeneity in survey methods, non-response rate, and context, it would be more appropriate to calculate this figure and update it regularly in each country.

The objectives of this study were to compare different estimates of alcohol availability in Spain during 2001–2011, build the best per capita consumption estimate (actual consumption), characterize trends over time, and quantify the extent to which actual consumption was approximated by other estimates, especially those based on self-reported data (coverage).

## Methods

### Data collection

Aggregate data were extracted from multiple sources. The Spanish Tax Agency (Tax Agency), Eurostat, World Health Organization (WHO), and Food and Agriculture Organization (FAO) provided data on alcohol sales/supplies; specifically, volumes or quantities of alcoholic beverages (those with >1.2 % alcohol-by-volume or ABV) available or intended for direct human consumption or to be drunk within Spain through legal distribution channels (alcohol availability). According to metadata included in the WHO data repository on recorded per capita alcohol consumption, Spanish WHO data for most of the study period were based on sales and taxation data, so they probably came ultimately from the Tax Agency. However, WHO and the Tax Agency were considered separately, because the immediate specific source of each WHO annual estimate was not explicit. Thus, there may have been intermediate Spanish administrative bodies between the Tax Agency and WHO that were involved in the preparation and submission of data, which in turn may have been inconsistent over time. The Spanish Food Consumption Panel of the Agriculture Ministry (PCA) provided data on the direct demand for alcohol; specifically, self-reported volume of alcoholic beverages purchased at retail within Spain for off- and on-premises consumption (self-reported purchases). The main data related to international visitors came from the Spanish Tourism Institute (i.e., arrivals and departures of tourists and same-day visitors, tourists’ length of stay), WHO (i.e., recorded per capita alcohol consumption by country), Eurostat, World Bank, and the scientific literature (Additional file 1). Other aggregate data were retrieved from the scientific literature; specifically, some data to estimate unrecorded alcohol consumption (alcohol consumed by Spanish residents but not included in routine statistics – such as smuggled and surrogate alcohol from informal sales or production, from products with ≤1.2 % ABV, and consumed/purchased by Spanish residents visiting abroad), alcohol losses (alcohol spilled, spoiled, or wasted, e.g., unfinished drinks, or used for cooking or purposes other than direct human consumption), and the alcohol consumed/purchased by foreign visitors in Spain [2, 13, 14, 18, 20–22].

Finally, Spanish National Health Surveys (ENS) and the European Health Survey in Spain provided individualized self-reported data on quantity and frequency (QF) of alcoholic beverages consumed anywhere in the last 12 months by household residents in Spain aged ≥15 (self-reported consumption) (Additional file 2). However, the Household Survey on Alcohol and Drugs in Spain (EDADES) could not be used because it only includes people aged 15–64.

### Data analysis

First, beverage categories and units of alcohol were homogenized. Beverages were stratified as beer, wine, and other beverages; the latter heterogeneous category including spirits or distilled beverages, aperitifs, or intermediate products (beverages 1.2–22 % ABV other than fermented ones) and cider, due to differences between sources in beverage categorization. Details are included in Additional file 1. Alcohol amount was always expressed in liters of pure alcohol per person-year (lpa/py), using official mid-year populations. Beverage mass was converted into volume by dividing by beverage density. Following WHO recommendations [1], beverage volumes were converted into lpa by applying percentages of ABV proposed by the Tax Agency: beer, cider, and wine-based mixtures (5.5 %), wine (11.5 %), aperitifs (15 %), and spirits (35 %) [23]. Between-agency variability or discrepancy in alcohol availability was measured using the between-agency range (difference between the highest and lowest estimate) and the between-agency coefficient of variation (CV) calculated as the ratio of the standard deviation to the mean multiplied by 100 [24]. This is a dimensionless measure of relative discrepancy, which allows comparisons of the between-agency discrepancy of different estimates of alcohol availability (i.e., by calendar year or beverage category) regardless of the estimate size.

To obtain actual alcohol consumption, a multistage process starting from the Tax Agency availability was followed. Tax Agency availability was estimated by adding the alcohol from beverage sales subject to excise duty on alcohol (beer, spirits, and aperitifs) and the alcohol from wine purchases self-reported to PCA. Thus, it does not include cider, and it is likely that self-reported wine purchases represent an important underestimation of wine sales, as in 2001–2011 self-reported purchases of beer and other beverages underestimated Tax Agency recorded sales by 43 and 45 %, respectively. Therefore, a multisource availability indicator was built by replacing the wine component in Tax Agency availability with the Eurostat wine supply and adding the FAO cider supply. Finally, actual consumption was obtained by adding the alcohol consumed/purchased abroad by residents in Spain and other unrecorded alcohol consumption, and subtracting alcohol losses and the alcohol consumed/purchased in Spain by foreign visitors (Additional files 3, 4, and 5). A sensitivity analysis was made varying assumptions in the calculation algorithms.

Self-reported consumption was obtained from individualized data on alcoholic beverages using the basic QF approach [25] (Additional file 2). Given the scarcity of updated empirical data [26], the alcohol content in standard drinks in Spain was estimated by applying the above mentioned percentages of ABVs to standard drink volumes in the high range of those included in drinking guidelines [27, 28]; the resulting amount of pure alcohol in grams (g) was 10.9 (beer, cider), 11.4 (wine), 11.8 (aperitifs), and 16.6 (spirits, spirits cocktails). A standard drink of local beverages was assumed to contain 10 g. A sensitivity analysis was performed by applying the alcohol contents suggested in ENS reports: 10, 10, 20, 20 and 10 g for beer, cider, aperitifs, spirits, and local beverages, respectively [29]. Survey estimates were weighted to account for strata oversampling or imbalance according to sex, age group, region, household size, nationality (Spanish/foreigner), and response rate. Confidence intervals at 95 % (95 % CI) were calculated accounting for the sampling design effect, which was calculated for the 2011 survey and applied to correct sampling variances in other surveys.

Coverage of Tax Agency availability, self-reported purchases and self-reported consumption was calculated as a ratio, with actual consumption as denominator and expressed as a percent. To identify linear time trends and significant changes in trends (joinpoints), various joinpoint regression models were used, allowing calculation of the average annual percent change (AAPC) for the entire period 2001–2011 and, if joinpoints, the annual percent change (APC) for each linear segment. AAPC and APC express the intensity and direction of the trend [30].

## Results

### Between-agency comparison in alcohol availability

There were significant decreases during 2001–2011 in total alcohol availability as calculated from Tax Agency and FAO data (AAPC range: −3.1–2.4), but not in that from WHO (AAPC = −0.9). There were significant decreases in wine availability from all agencies (APPC range: −8.0–4.8), upward or stable trends for beer availability (APPC range: 0.4–1.5), and mixed trends for other beverages. Tax Agency and WHO estimates were almost the same for wine in each year of the period, and for beer and other beverages from 2005 onwards.

**Fig. 1:**
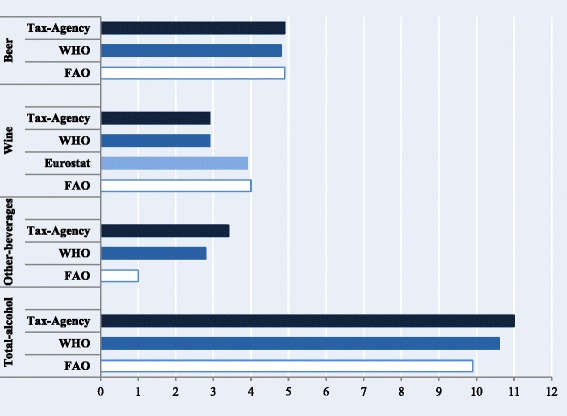
Per capita alcohol availability by agency and beverage category, Spain, 2001–2011 (Liters of pure alcohol/person-year). Footnotes: *Other beverages* include spirits, cider, and aperitifs (beverages 1.2–22 % alcohol-by-volume other than fermented ones, such as vermouth, sherry or port). *Tax Agency* Spanish Tax Agency, *WHO* World Health Organization, *FAO* Food and Agriculture Organization

A considerable between-agency discrepancy in estimates was observed. Thus, the between-agency range for total alcohol was 1.2 and 1.3 lpa/py, respectively, in 2001–2011 and 2011. By beverage category, the range of estimates was very small for beer (0.1 lpa/py), while it was large for wine (1.1 lpa/py), and especially for other beverages (2.3 lpa/py) – in the first case, due to low Tax Agency and WHO estimates, and in the second, to very low FAO estimates (Table 1, Fig. 1). Regarding trends, during 2001–2011, the joinpoint analysis shows significant downward linear trends in the between-agency range for other beverages (AAPC: −4.5) and total alcohol (AAPC: −9.5). Moreover, for wine, a joinpoint from a non-significant upward trend towards a non-significant downward trend was identified at 2006.

**Table 1:** Alcohol availability estimates from different agencies and between-agency range by beverage category, Spain, 2001–2011

Agency	Beverage	Year												AAPC_2001–2011_ (95 % CI)
		2001	2002	2003	2004	2005	2006	2007	2008	2009	2010	2011	2001–11	
		Alcohol availability (liters of pure alcohol/person-year)												
Tax Agency	Beer	4.7	4.6	4.9	4.9	4.9	5.1	5.1	4.9	4.9	4.8	4.9	4.9	0.4 (−0.5,1.3)
	Wine	4.0	3.8	3.6	3.6	3.4	3.2	2.5	2.2	2.1	2.0	1.8	2.9	−8.0 (−10.4, −5.6)
	Other beverages	4.5	3.1	3.7	4.0	3.5	3.5	3.4	3.1	2.9	2.9	2.7	3.4	−3.7 (−5.6, −1.7)
	Total alcohol	13.1	11.5	12.2	12.6	11.8	11.8	11	10.2	9.9	9.7	9.4	11.1	−3.1 (−4.0, −2.1)
World Health Organization	Beer	4.4	4.2	4.5	4.5	5.0	5.2	5.1	4.9	5.0	4.9	4.9	4.8	1.5 (0.1,3.0)
	Wine	3.9	4.0	3.7	3.8	3.4	3.2	2.6	2.2	2.1	2.0	1.8	2.9	−7.9 (−10.4, −5.3)
	Other beverages	2.8	1.8	2.0	2.1	3.5	3.5	3.4	3.1	2.9	2.9	2.7	2.8	3.3 (−1.4,8.2)
	Total alcohol	11.1	9.9	10.2	10.4	11.9	11.9	11.1	10.2	10.0	9.8	9.4	10.6	−0.9 (−3.4,1.6)
Eurostat	Wine	4.8	4.5	4.4	4.4	4.3	4.1	4.0	3.6	3.2	3.0	3.0	3.9	−4.8 (−6.2, −3.4)
Food and Agriculture Organization	Beer	4.6	4.7	5.0	5.1	4.8	5.1	5.1	4.9	4.9	4.8	4.8	4.9	0.5 (−0.8, 1.7)
	Wine	4.7	4.5	4.8	4.9	4.7	4.9	3.6	3.9	2.9	3.2	2.3	4.0	−6.3 (−10.1, −2.4)
	Other beverages	1.1	1.1	1.1	1.1	1.0	1.0	1.0	1.0	1.0	1.0	1.0	1.0	−1.2 (−1.8, −0.6)
	Total alcohol	10.4	10.3	11.0	11.0	10.5	11.0	9.7	9.8	8.8	9.0	8.1	9.9	−2.5 (−4.0, −0.9)
		Between-agency range in alcohol availability (liters of pure alcohol/person-year)												
	Beer	0.2	0.5	0.6	0.6	0.2	0.0	0.1	0.0	0.1	0.1	0.0	0.1	
	Wine	0.9	0.7	1.3	1.3	1.2	1.7	1.4	1.6	1.1	1.3	1.2	1.1	
	Other beverages	3.4	2.0	2.6	2.9	2.5	2.5	2.4	2.1	1.9	2.0	1.7	2.3	
	Total alcohol	2.8	1.6	1.9	2.1	1.4	0.8	1.3	0.5	1.2	0.8	1.3	1.2	

Liters of pure alcohol/person-year: Calculated by multiplying the annual beverage volume in liters by the ABV percentage of each beverage [beer, cider, wine-based mixtures (5.5 %), wine (11.5 %), aperitifs (15 %), and spirits (35 %)] and dividing by official mid-year population aged ≥15. AAPC_2001–2011_: Average annual percent change during 2001–2011 from joinpoint regression

Tax Agency: “Other beverages” includes spirits and aperitifs (beverages 1.2–22 % ABV other than fermented ones, such as vermouth, sherry, or port). Beer, spirits, and aperitif estimates were calculated by dividing the receipts from excise duties on each beverage between the weighted average tax rate applicable, and wine estimate was obtained directly from purchases self-reported to the Spanish Food Consumption Panel

Word Health Organization: Includes “recorded alcohol” figures from the Global Information System on Alcohol and Health. “Other beverages” includes spirits, aperitifs, and cider. 2011 data were imputed from the Spanish Tax Agency

Food and Agriculture Organization: “Wine” includes wine and aperitifs. “Other beverages” includes spirits and cider

Between-agency range was obtained as the difference between the highest and lowest agency estimate of alcohol availability

**Fig. 2:**
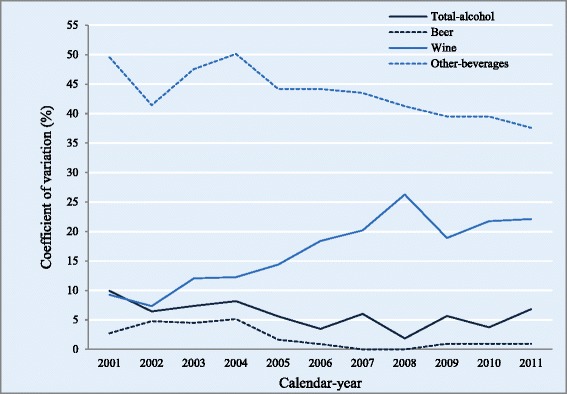
Between-agency coefficient of variation for per capita alcohol availability by beverage category and calendar year, Spain, 2001–2011. Footnotes: The *coefficient of variation (CV)* was calculated as follows: CV = (σ/μ)100, where σ was the between-agency standard deviation and μ the arithmetic mean of per capita alcohol availability. σ was calculated as σ= $\sqrt{\frac{\left[{\sum \left({\mathrm{x}}_i-\upmu \right)}^2\right]}{\mathrm{N}}}$, where x_i_ is each agency estimation and N is the number of agencies providing each estimation. *Other beverages* include spirits, cider, and aperitifs (beverages 1.2–22 % alcohol-by-volume other than fermented ones, such as vermouth, sherry, or port)

The between-agency CV in the entire period 2001–2011 was 4.8 % (total alcohol), 1.0 % (beer), 15.0 % (wine), and 41.5 % (other beverages). CVs by calendar year and beverage category are shown in Fig. 2. Regarding trends, the joinpoint analysis shows significant linear trends in CV for other beverages (AAPC: −2.3), and wine (APPC: 10.0), although for wine, a joinpoint from a significant upward trend towards a non-significant downward trend was identified at 2008. The downward trend for total alcohol did not reach statistical significance.

### Self-reported purchases and self-reported consumption

Self-reported alcohol purchases and consumption were 6.3 lpa/py and 2.6 lpa/py (95 % CI: 2.6, 2.7), respectively, in 2011. Self-reported purchases showed significant decreases for total alcohol, beer and wine; joinpoints were identified at 2004, with significant decreases in subsequent years. Significant decreases were also observed in self-reported consumption for total alcohol, beer, and wine, and these were much stronger than for self-reported purchases (Table 2). By using the standard drink units proposed in ENS reports, similar self-reported consumption estimates in 2011 were obtained (2.5 lpa/py; 95 % CI: 2.5, 2.6).

**Table 2:** Multisource alcohol availability, self-reported purchases, and self-reported consumption by beverage category, Spain, 2001–2011

	Year												AAPC_2001–2011_ (95 % CI)	Joinpoint identified	
Beverage	2001	2002	2003	2004	2005	2006	2007	2008	2009	2010	2011	2001–11			
														AAPC_2001–2006_ (95 % CI)	AAPC_2006–2011_ (95 % CI)
	Multisource alcohol availability (Liters of pure alcohol/person-year)														
Beer	4.7	4.6	4.9	4.9	4.9	5.1	5.1	4.9	4.9	4.8	4.9	4.9	0.4 (−0.5,1.3)	1.7 (0.2,3.3)	−1.0 (−2.5,0.6)
Wine	4.8	4.5	4.4	4.4	4.3	4.1	4	3.6	3.2	3.0	3.0	3.9	−4.8 (−6.2, −3.4)	−2.5 (−5.0, −0.1)	−7.0 (−9.4, −4.6)
Other beverages	4.6	3.2	3.7	4.0	3.5	3.5	3.4	3.1	2.9	3.0	2.8	3.4	−3.6 (−5.4, −1.7)	_	_
Total alcohol	14.0	12.3	13	13.3	12.7	12.8	12.5	11.6	11.1	10.8	10.6	12.2	−2.4 (−3.3, −1.5)	_	_
	Self-reported alcohol purchases (Liters of pure alcohol/person-year)													AAPC_2001–2004_ (95 % CI)	AAPC_2004–2011_ (95 % CI)
Beer	2.9	2.8	3.0	3.1	3.0	3.0	2.7	2.6	2.6	2.5	2.4	2.8	−1.6 (−3.0, −0.2)	3.1 (−2.0,8.4)	−3.6 (−4.9, −2.3)
Wine	4.1	3.9	3.7	3.8	3.5	3.3	2.9	2.5	2.3	2.2	2.1	3.1	−6.7 (−8.4, −4.9)	−2.2 (−8.5,4.5)	−8.5 (−10.2, −6.9)
Other beverages	2.1	1.8	1.9	1.9	1.8	1.8	1.9	1.9	1.8	1.8	1.8	1.9	−0.8 (−1.7,0.1)	_	_
Total alcohol	9.1	8.6	8.6	8.8	8.3	8.1	7.5	7.0	6.7	6.4	6.3	7.7	−3.6 (−4,6, −2.6)	−0.8 (−4.2,2.8)	−4.8 (−5.7, −3.9)
	Self-reported alcohol consumption (Liters of pure alcohol/person-year)														
Beer	2.2					1.4			1.3		1.1	1.5	−6.2 (−10.7, −1.4)		
Wine	2.7					1.7			1.1		1.0	1.6	−9.9 (−13.5, −6.1)		
Other beverages	1.4					0.7			0.7		0.5	0.8	−8.5 (−18.2,2.3)		
Total alcohol	6.3					3.8			3.0		2.6	3.9	−8.4 (−10.6, −6.1)		

Liters of pure alcohol/person-year: Calculated by multiplying the annual beverage volume in liters by the percent ABV of each beverage [beer, cider, wine-based mixtures (5.5 %), wine (11.5 %), aperitifs (15 %), and spirits (35 %)] and dividing by official mid-year population aged ≥15

Other beverages: Spirits, aperitifs, and cider

Multisource alcohol availability: Wine figures from the Spanish Tax Agency were replaced by Eurostat wine retail supplies, and FAO cider supplies were added

Self-reported alcohol purchases: Self-reported alcoholic beverages purchased at retail within Spain for off- and on-premises consumption, from the Spanish Food Consumption Panel. Wine-based mixtures were grouped with wine

Self-reported alcohol consumption: Based on individualized data from Spanish National Health Surveys (2001, 2006 and 2011) and the European Health Survey in Spain (2009), assuming a certain volume for the standard drink of each beverage category in the survey (See details in Additional file 2)

AAPC_2001–2011_ (95 % CI): Average annual percent change (confidence interval at 95 %) for 2001–2011 from joinpoint regression

APC (95 % CI): Annual percent change (confidence interval at 95 %) for the period in subscript when a joinpoint was identified

### Estimating multi-source availability and actual per capita consumption

Multi-source availability for total alcohol was 10.6 lpa/py in 2011, with significant decreases observed for total alcohol, wine, and other beverages in 2001–2011; joinpoints were identified at 2006 which showed an increasing trend followed by stability in the case of beer, and acceleration of the decreasing trend for wine (Table 2). Estimates for unrecorded alcohol, alcohol losses, and alcohol consumed/purchased by Spanish residents visiting abroad and foreign visitors in Spain were, respectively, 0.6, 0.9, 0.3, and 1.1 lpa/py (Additional file 4). The latter estimate represented 9.8 % of alcohol consumed/purchased in Spain in 2011.

**Fig. 3:**
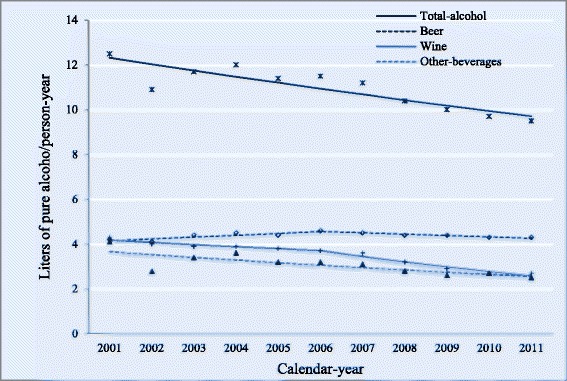
Trends in actual per capita alcohol consumption modeled with joinpoint regression by beverage category, Spain, 2001–2011. Footnotes: Data markers (*asterisks*, *diamonds*, *crosses*, and *triangles*) represent series of observed data (total alcohol, beer, wine, and other beverages), while the corresponding *lines* represent series of modeled data. 2006 joinpoints for beer and wine express statistically significant trend changes. *Other beverages* include spirits, cider, and aperitifs (beverages 1.2–22 % alcohol-by-volume other than fermented ones, such as vermouth, sherry, or port)

Actual per capita consumption in 2011 under intermediate assumptions was 9.5 lpa/py, ranging from 8.6 to 10.4 lpa/py under different assumptions. Significant decreases in intermediate estimates of actual consumption were observed in 2001–2011 for total alcohol (AAPC: −2.3), wine (AAPC: −4.6), and other beverages (AAPC: −3.5). Joinpoints were identified at 2006 from increasing to stability (beer) and from stability to decreasing (wine) (Table 3, Fig. 3). These trends resulted in changes in the beverage-specific contribution to total alcohol consumption between 2001 and 2011 from 33 to 46 % (beer), 34 to 28 % (wine), and 33 to 26 % (other beverages).

**Table 3:** Actual per capita alcohol consumption and coverage of different indicators by beverage category, Spain, 2001–2011

	Year														
	2001	2002	2003	2004	2005	2006	2007	2008	2009	2010	2011	2001–2011	AAPC_2001–2011_ (95 % CI)	Joinpoint identified	
														APC_2001–2006_ (95 % CI)	APC_2006–2011_ (95 % CI)
	Actual per capita alcohol consumption (Liters of pure alcohol/person-year)														
Beer	4.2	4.1	4.4	4.5	4.4	4.6	4.5	4.4	4.4	4.3	4.3	4.4	0.3 (−0.6,1.2)	1.9 (0.3,3.5)	−1.3 (−2.9,0.2)
Wine	4.3	4	3.9	3.9	3.8	3.7	3.6	3.2	2.9	2.7	2.7	3.5	−4.6 (−6.0, −3.2)	−2.4 (−4.9,0.2)	−6.9 (−9.2, −4.4)
Other beverages	4.1	2.8	3.4	3.6	3.2	3.2	3.1	2.8	2.6	2.7	2.5	3.1	−3.5 (−5.5, −1.5)	_	_
Total alcohol	12.5	10.9	11.7	12	11.4	11.5	11.2	10.4	10	9.7	9.5	10.9	−2.3 (−3.3, −1.4)	_	_
	Coverage of Tax Agency availability (%)														
Beer	111.5	112.7	111	111.1	112	111.7	111.6	111.5	111.5	111.3	112.3	111.6	−0.0 (−0.1,0.1)		
Wine	92.8	94.2	90.9	92.8	89.8	85.9	71.1	69.1	71.7	73.4	69	82.8	−3.6 (−4.8, −2.3)		
Other beverages	110.5	111.1	109.6	109.7	110.4	110.2	110	109.7	109.8	109.4	110.4	110	−0.1 (−0.2, −0.0)		
Total alcohol	104.8	105.4	103.8	104.7	104.1	102.9	98.2	97.9	99.5	100.2	99.5	101.9	−0.7 (−1.1, −0.4)		
	Coverage of self-reported alcohol purchases (%)													APC_2001–2004_ (CI95%)	APC_2004–2011_ (CI95%)
Beer	69.5	70	67.5	69.6	67.7	64.8	60.3	59.6	58.6	57.3	54.5	63.3	−2.4 (−3.3, −1.5)	−0.3 (−3.5,2.9)	−3.3 (−4.1, −2.4)
Wine	95.5	96.8	93.7	95.7	92.5	88.7	80.1	77.9	78.6	80	80.1	87.9	−2.4 (−3.3, −1.6)	_	_
Other beverages	50.8	65.2	57.6	52.7	57.3	58.2	61.6	69.4	68.8	66.1	71.9	61.1	2.7 (1.0,4.4)	_	_
Total alcohol	72.2	78.6	73.4	73.1	73.1	70.7	67	67.9	67.1	66.1	66.3	70.5	−1.5 (−2.1, −0.9)	_	_
	Coverage of self-reported alcohol consumption (%)														
Beer	53.1					30.9			28.4		26.3	34.1	−6.8 (−12.5, −0.8)		
Wine	63.1					45.3			38.2		36.9	47.5	−5.4 (−7.9, −2.8)		
Other beverages	34.5					23.6			25.7		21.4	26.8	−4.2 (−10.1,2.2)		
Total alcohol	50.5					33.6			30.5		28	36.2	−5.7 (−9.1, −2.1)		

Actual per capita alcohol consumption: Alcohol consumed by the population resident in Spain aged ≥15 (*C*_*P*_) under intermediate assumptions. *C*_*P*_ *= R + C*_*SV+*_ *I*_*SV*_ *+ U-L-C*_*FV*_ *-E*_*FV*_; *U = U*_*T*_ *-C*_*SV*_ *-I*_*SV*_, where *R* is multisource availability, *C*_*SV*_ is alcohol consumed abroad by Spanish visitors, *I*_*SV*_ is alcohol purchased abroad by Spanish visitors and personally imported to Spain, *U* is unrecorded alcohol other than *C*_*SV*_ and *I*_*SV*_, *L* is alcohol lost after sale, *C*_*FV*_ is alcohol consumed in Spain by foreign visitors, *E*_*FV*_ is alcohol purchased in Spain by foreign visitors and personally exported from Spain, and *U*_*T*_ is total unrecorded alcohol. See details in Additional file 3. Assumed parameters values for intermediate estimate were: Souvenir factor (*S*) for foreign visitors to Spain and Spanish visitors abroad = 0.2 lpa/visitor; Holiday factor (*H*) for foreign visitors to Spain and Spanish visitors abroad = 1.25; Price elasticity of demand (*E*_*d*_) = 0.50; *U*_*T*_ = 9 % of *R*, with a relative decline of 1 % annually, *L* = 8 % of (*R + C*_*SV*_ *+ I*_*SV*_ *+ U*)

Coverage (%): Each indicator divided by actual consumption and multiplied by 100

Other beverages: Spirits, aperitifs, and cider

AAPC_2001–2011_ (95 % CI): Average annual percent change (confidence interval at 95 %) for 2001–2011 from joinpoint regression

APC (95 % CI): Annual percent change (confidence interval at 95 %) for the period in subscript when a joinpoint was identified

### Coverage of selected indicators compared to per capita consumption

In 2011 coverage of Tax Agency availability, self-reported purchases and self-reported alcohol consumption compared to the intermediate estimate of actual consumption was, respectively, 99.5, 66.3, and 28.0 % (95 % CI: 27.1, 28.9), with significant decreases over time in the coverage of the three indicators, especially in the latter. Regarding beverages, coverage of Tax Agency availability showed significant decreases for wine, coverage of self-reported purchases for wine and beer, and coverage of self-reported consumption for all beverage categories. Moreover, coverage of self-reported purchases showed a significant increase for other beverages. A joinpoint at 2004 from stability toward a downward trend was observed for beer purchases. Self-reported indicators (purchases and consumption), showed higher coverage for wine than for beer and other beverages, while the opposite occurred with Tax Agency availability (Table 3).

WHO availability coverage was 99.5 % in 2011, showing a significant increase over time. Multi-source availability overestimated actual per capita consumption by 12.3 % (range: 2.5–24.1 %). In sensitivity analyses using standard drink units proposed in ENS reports, self-reported consumption coverage for total alcohol, wine, beer, and other beverages was 26.8, 32.5, 24.2, and 25.1 %, respectively. Considering both random variability (95 % CI) in the numerator and the uncertainty of assumptions derived from sensitivity analyses in numerator and denominator, the self-reported consumption coverage for total alcohol would range from 23.8 to 32.0 %.

## Discussion

### Main findings

Between-agency discrepancy in alcohol availability estimates remained substantial in 2011, mainly because of other beverages (primarily spirits) and wine, although some decrease could be observed during 2001–2011. Actual per capita consumption was 9.5 l of pure alcohol per person-year in 2011, decreasing 2.3 % annually in 2001–2011, mainly due to decreases in wine and other beverages. The coverage of WHO availability, Tax Agency availability, self-reported purchases, and self-reported consumption compared to actual consumption was, respectively, 99.5, 99.5, 66.3, and 28.0 % in 2011, with downward time trends in the last three estimates, especially for self-reported consumption, and an upward trend in WHO availability. Multisource availability involved an overestimation of 12.3 %, mainly due to tourism imbalance.

### Considerable between-agency discrepancy in alcohol availability

The study shows that in 2011 the between-agency discrepancy in per capita alcohol availability estimates remained high. Thus, the between-agency range and CV for total alcohol were 1.3 lpa/py, and 6.8 %, respectively. This probably was mainly due to excessively low estimates for wine (Tax Agency and WHO) and spirits (FAO). Important discrepancies may also exist elsewhere [1]. In fact, in 2010 in France, Italy, Greece, and Portugal, the between-agency CVs of availability estimates considering WHO, FAO, and Eurostat varied between 3.5 and 14.8 % (total alcohol), 3.2–12.4 % (beer), 3.0–19.0 % (wine) and 8.7–45.6 % (other beverages). This situation is worrisome because alcohol availability is often the standard indicator in spatiotemporal comparisons of alcohol consumption [1, 31].

The absolute discrepancy in total alcohol availability (as measured by the range) showed an average annual decline of 9.5 % during 2001–2011, although this decline is partly explained by the declining availability. Thus, when CV was used as the measure of relative discrepancy, the decrease was not statistically significant for total alcohol, although it was for other beverages.

### Clear decrease in per capita alcohol consumption

Using uncorrected figures of alcohol availability from individual agencies to derive temporal trends in per capita alcohol consumption in Spain may be problematic. Thus, during 2001–2011 significant decreases in estimates of total alcohol availability from the Tax Agency and FAO, but not from WHO, were identified. Failure to identify a significant decrease in WHO availability (“recorded alcohol consumption”) was likely due to changes in data sources or data collection procedures. In fact, Tax Agency and WHO figures for total alcohol were very similar from 2005 onwards, suggesting that the WHO data source had changed from that year on. Mixed trends by agency in the availability of beer and other beverages were also identified.

Correcting alcohol availability by triangulating different sources (multisource availability) provides clear trends in per capita alcohol consumption, which decreased by 2.3–2.4 % annually during 2001–2011, prolonging the pattern started around 1975. Declines have been also observed in most European countries [22, 32], and could be due to demographic, socioeconomic (including recession and unemployment), and cultural changes, as well as the possible effectiveness of some interventions [13, 33, 34]. Unfortunately, there is little empirical evidence on the quantification of each of these factors. Population aging during the period was negligible. Instead, the increase in immigrants’ share of Spain’s residents (5.3 % in 2001 and 13.5 % in 2011) may have had some relevance, given that most immigrants in Spain drink less than natives [35], and the country’s progressive urbanization [4] (Spanish people living in cities >50,000 inhabitants increased from 47 % in 2001 to 52 % in 2011) may also have had an effect.

The role of economic factors is uncertain. Before the crisis starting in 2007–2008, alcohol consumption decreased, while alcohol affordability remained stable or increased [4, 36]. However, the identification of joinpoints at 2006 for beer and wine (toward stability and decreasing, respectively), suggests that the crisis could have contributed to the decline in consumption, affecting mostly drinking on premises, which continues to represent the most important component of consumption in Spain [37]. A recent study suggests that during the economic recession in Spain, regular excessive drinking decreased while binge drinking increased [38]. Finally, some interventions may have been effective, such as advertising regulations, increasing the minimum drinking age, workplace drinking bans, and traffic safety policies [34]. However, risk perception of drinking did not increase [39]. Consumption peaks in 2001 and 2004 were probably artifacts due mainly to stockpiling of spirits, triggered by rumors of possible tax increases [23], which hardly affected retail purchases. A progressive change of the dominant beverage from wine to beer was observed from the beginning of the study period [4], so that in 2011 beer accounted for 46 % of total alcohol consumption vs. 28 % for wine.

Although homogenizing trends in drinking patterns across Europe have been suggested [13], probably due to drinking patterns of youth, in other traditionally wine-drinking countries, such as France, Italy, Portugal, or Greece, wine remains the dominant beverage [22, 40].

### Hidden overestimation of per capita consumption by alcohol availability

The multisource availability figures clearly overestimated actual consumption in Spain (by 12.3 % in 2011 under intermediate assumptions). This is mainly due to the consumption/purchases imbalance between foreign visitors in Spain and Spanish visitors abroad (tourism imbalance). Thus, in 2011 in Spain there was an excess of 56.9 million international visits and 412.4 million international overnights, and most foreign visitors came from European countries with higher alcohol prices than Spain [22, 41]. In fact, without tourism imbalance, overestimation would have been only 2.2 %. Such an imbalance could also have an important effect on actual alcohol consumption in other countries, although the direction of the effect depends on the predominance of inbound or outbound international visits [1]. In Spain, correction of the Tax Agency availability indicator to obtain the multisource availability was a methodological need due to underestimation of wine and cider sales/supplies by the tax agency, an underestimation that also transferred to the WHO availability figures starting in 2005 (which is generally referred to as “recorded per capita alcohol consumption”). In the absence of this correction, the similarity between coverage of the Tax Agency and WHO availability (≈100 % in 2011) gives an apparent coherence to these indicators and spuriously supports their validity.

### Poor and decreasing coverage of per capita consumption by self-reported behaviors

Self-reported consumption coverage in Spain (28.0 % in 2011; range: 23.8–32.0 %) seems lower than elsewhere (30–65 %) [1, 7, 9, 14, 16–19], although figures outside this range have been reported [42–44].

Consumption by people aged <15 does not explain the low coverage because, as reported elsewhere [10], analysis of Spanish school surveys [39] suggested it was negligible.

Consequently, the low coverage must be attributed to underestimation of consumption by population surveys, affecting mainly heavy and binge drinking [45, 46]. This could be due to various factors [1, 14, 25], including social desirability bias [47], difficulty in quantifying and averaging consumption (especially if questions on frequency of drinking are not sufficiently disaggregated by beverage) [17], high probability of sampling exclusion of heavier drinkers due to homelessness/housing instability or living in communal establishments (student’s group quarters, prisons, hostels, etc.) or high consumption periods like Christmas or summer holidays [48, 49], and assumption of standard drink volumes lower than commonly used, especially for free-poured beverages, etc. [50–54]. Moreover, the varying alcohol content in emerging products [55, 56] complicates data collection.

Given the low coverage of self-reported consumption, using uncorrected figures to estimate alcohol-attributable disease burden or prevalence of heavy drinkers [57] will greatly underestimate alcohol-related problems and intervention needs. Considering the highest range of estimated coverage, in Spain self-reported consumption figures in 2011 should be shifted upwards by as much as 91 % of any estimates of WHO or Tax Agency availability.

Self-reported purchases coverage was 66.3 % in 2011. Higher coverage (87–90 %) has been found in Sweden and UK [9, 10]. Underestimation of purchases can be due to incomplete recording by panelists (mostly affecting small and irregular purchases), failure to consider certain beverages (i.e., alcopops), sampling bias (e.g., failure to consider off-premise alcohol purchases, made outside the household frame usually by youth) or even alcohol losses between wholesale and retail markets. The observed lower underestimation compared to self-reported consumption could be explained by less reluctance to report purchases than consumption, and less difficulty in remembering and summarizing quantities of bottles/cans purchased than multiple drinking events, especially if purchases are recorded by automatic barcode reading [9].

Downward trends in coverage of self-reported indicators were observed in 2001–2011. The decline in self-reported consumption coverage (5.7 % annually) was especially intense, and is explained by the faster decline in self-reported consumption (8.4 % annually) compared with actual consumption (2.4 % annually). Large declines in age-adjusted prevalence of risk drinkers based on ENS self-reported consumption have also been found [58]. However, the decline during 2001–2011 in self-reported consumption from the EDADES survey [39] was much smaller (1.5 %).

A good spatiotemporal correlation between self-reported alcohol consumption and availability has been found elsewhere [1, 43]. However, declines in self-reported consumption coverage have been also observed; it has been suggested these may be partly due to the growing contribution to total alcohol consumption of binge drinking and irregular heavy drinking patterns, which are poorly captured by the usual basic QF approach [25, 49]. This is also plausible in Spain where the prevalence of these patterns is increasing [59–61]. However, the decline in ENS coverage is probably due mainly to the fact that the instruments used in the most recent surveys do not capture consumption as well as those previously used. The main reasons are that the decline is very strong, and that coverage in the EDADES Survey, which has had more consistent data collection over time [61], has not fallen (31.1 % in 2001 and 36.2 % in 2011).

Finally, a probable increase in the proportion of off-premise alcohol purchases outside the household frame (not collected) may have contributed to decreasing coverage of self-reported purchases. The joinpoint at 2004 toward a decline in beer purchases coverage is probably an artifact caused by the exclusion of alcohol-free beer starting in that year. By beverage, the highest coverage was found for wine and the lowest for other beverages, both in self-reported purchases and consumption, with heterogeneous findings elsewhere [9, 10].

### Strengths and limitations

Data from multiple sources were triangulated to obtain actual consumption for Spanish residents. This revealed the weakness of official national estimates, which is often transferred to international statistics. Calculation algorithms incorporate data on various factors affecting the estimates, especially regarding consumption/purchases during international visits (Spanish residents abroad and foreigners in Spain). However, empirical national data on various aspects were lacking. Thus, assumptions were made based essentially on international studies [4, 10, 13, 18, 21, 22, 62]. The algorithms and methodology used have been clearly explained, and the sensitivity analyses allowed calculation of uncertainty ranges for estimates. The main uncertainties arise in estimating unrecorded alcohol consumption and purchases during international visits. Estimates of unrecorded alcohol have little empirical basis; in Spain, its main component is probably derived from informal winemaking, although this should have declined with progressive urbanization. Smuggled or surrogate alcohol is likely to be negligible due to low alcohol prices. As for purchases during international visits, the algorithms combine empirical data (i.e., number of visits, average stay, the ratio between Spain and foreign countries of alcohol prices, and of per capita alcohol consumption) and assumptions (price elasticity of alcohol demand [36], or average quantity of alcohol purchased in a visit involving countries with alcohol prices and per capita consumption similar to Spain) (Additional files 3, 4, and 5). Moreover, the number of entries and overnight stays of foreigners in Spain might be somewhat higher than estimated because some illegal immigrants cannot be included in the population register. However, the phenomenon probably has low relevance, because in 2001–2011 very few conditions were required to be included in this register [35]. Finally, identical alcoholic beverage strength (ABV percentage) was applied throughout the entire period, but as elsewhere, changes over time cannot be ruled out, especially affecting wine strength, which might have increased [63–65].

## Conclusions

This work shows the weakness of existing per capita alcohol consumption estimates in Spain during 2001–2011. Substantial between-agency discrepancies in alcohol availability have been found, which probably also exist in many countries. The quality and consistency of alcohol availability data should be improved, establishing greater coordination among agencies and seeking consensus on data management protocols. Meanwhile, triangulation of availability data from various sources seems essential. The availability of alcoholic beverages not subject to excise duty appears to be better reflected in non-financial balance sheets than in self-reported purchases. Directly taking uncorrected alcohol availability, and especially self-reported purchases or consumption, to derive time trends in actual alcohol consumption can lead to misjudgments in assessing intervention needs and effectiveness. In Spain, the multiplier factor to shift self-reported consumption figures upward to obtain conservative estimates of alcohol-attributable disease burden or heavy drinking prevalence should be somewhat higher than elsewhere due to surplus tourism. The QF instrument currently used in population surveys in Spain, derived from the European Health Interview Survey, seems to greatly underestimate actual per capita alcohol consumption. It should be complemented with appropriate questions to better characterize binge drinking and irregular drinking patterns.

Extensive empirical work remains to be done to improve estimates of per capita alcohol consumption, focusing on alcohol consumption/purchases by international visitors, alcohol losses, unrecorded alcohol, volume and strength of standard drinks, and other aspects.

## Abbreviations

AAPC, average annual percent change; ABV, alcohol-by-volume or content of pure alcohol by beverage volume; APC, annual percent change; FAO, Food and Agriculture Organization; lpa/py, liters of pure alcohol per person-year; QF, quantity and frequency; ENS, Spanish National Health Survey; Tax Agency, Spanish Tax Agency; WHO, World Health Organization; 95 % CI, confidence intervals at 95 %.

## Additional files

Additional file 1: Characteristics of the main aggregate data regularly provided by different sources to estimate actual per capita alcohol consumption in Spain, 2001–2011. The definition and method of production of the main aggregate data regularly provided by different sources to estimate actual per capita alcohol consumption in Spain are described in detail, as well as how to access data and metadata. The main data sources considered are the Spanish Tax Agency, Eurostat, Word Health Organization, Food and Agriculture Organization, Spanish Food Consumption Panel, Spanish Tourism Institute, World Bank, and National Statistics Institute. (DOCX 17 kb) Additional file 2: Methodological characteristics of main population surveys providing data to calculate self-reported alcohol consumption among population aged ≥15, and calculation algorithms, Spain, 2001–2011. The characteristics considered are: year of the survey, effective sample size, mode of questionnaire administration, questions on frequency of consumption, questions on number of standard drinks, response rate, and percent of missing values for quantity-frequency questions. (DOCX 15 kb) Additional file 3: Algorithms and assumptions to estimate actual per capita alcohol consumption from multisource availability, Spain, 2001–2011. Definition, algorithms, and assumptions are included for the following indicators: actual per capita alcohol consumption, alcohol consumption abroad by Spanish visitors, alcohol imported by Spanish visitors abroad, other unrecorded alcohol, alcohol losses after sale, alcohol consumption in Spain by foreign visitors, and alcohol exported from Spain by foreign visitors. (DOCX 21 kb) Additional file 4: Results for the different indicators needed to estimate alcohol actual per capita alcohol consumption from multisource alcohol availability under various assumptions, 2001–2011. Liters of pure alcohol/person-year. The results for the main indicators needed to obtain actual per capita alcohol consumption from multisource alcohol availability in the period 2001–2011 are shown. Indicators considered are: alcohol consumed/purchased by Spanish visitors abroad, unrecorded alcohol, alcohol lost, and alcohol consumed/purchased by foreign visitors in Spain. Estimates were obtained under intermediate, high, and low assumptions. (DOCX 18 kb) Additional file 5: Example of estimation of alcohol consumed/purchased in visits to Spain by foreign residents and visits abroad by Spanish residents, 2011. Data for different countries on parameters needed for the estimation are included, specifically on: total alcohol per capita consumption, alcohol price index, number of inbound and outbound visits, and number of inbound and outbound stays. The calculations are disaggregated for overnight visitors and same-day visitors. (DOCX 24 kb)
