# Supplementary material for: Estimating average alcohol consumption in the population using multiple sources: the case of Spain
Source: Popul Health Metr. 2016 Jun 2;14:21. doi: 10.1186/s12963-016-0090-4 (PMC4890273; doi:10.1186/s12963-016-0090-4)
Supplement: Additional file 1: — Characteristics of the main aggregate data regularly provided by different sources to estimate actual per capita alcohol consumption in Spain, 2001–2011. The definition and method of production of the main aggregate data regularly provided by different sources to estimate actual per capita alcohol consumption in Spain are described in detail, as well as how to access data and metadata. The main data sources considered are the Spanish Tax Agency, Eurostat, Word Health Organization, Food and Agriculture Organization, Spanish Food Consumption Panel, Spanish Tourism Institute, World Bank, and National Statistics Institute. (DOCX 17 kb) [file 12963_2016_90_MOESM1_ESM.docx]

Additional file 1. Characteristics of the main aggregate data regularly provided by different sources to estimate actual per capita alcohol consumption in Spain, 2001-2011

| **Data source** | **Indicators: definition and extraction by source** | **Comments and access to data and metadata** |
| --- | --- | --- |
| Spanish Tax Agency (AEAT) | *Tax agency alcohol availability [Estimated alcohol consumption]:* Annual volume in millions of liters of beverages with >1.2% ABV released for direct human consumption within Spain. Categories: beer, wine, spirits, aperitifs. Cider is not included. The volume of beverages subject to excise duty (beer, spirits, aperitifs) is estimated by dividing the tax receipts of each beverage by the weighted average tax rate applicable. Wine volume is taken directly from FCP. Alcohol losses after release of beverages (transport, storage, distribution, consumption) are included^a^*.* | The tax is applied in the early stages of the marketing process (production, importation) and paid when the beverage is released from factories or tax warehouses or the customs debt is generated. Thus, tax agency availability generally represents wholesales.  Ref.: <http://www.agenciatributaria.es/AEAT.internet/Inicio/Aduanas_e_Impuestos_Especiales/Impuestos_Especiales/Impuestos_Especiales.shtml>. |
| Eurostat | *Eurostat wine availability [Gross human consumption of wine]*: Annual wine supplies in hectoliters available for human food use at retail level within Spain, extracted from supply balance sheets. It is calculated as wine produced plus imported plus stock variation minus that exported, processed, dedicated to nonfood uses, and lost during storage and transportation. | Producers' own consumption is included. Losses and variations in stocks refer to institutional and consumer levels.  Ref.: <http://ec.europa.eu/eurostat/web/agriculture/data/database> |
| Word Health Organization (WHO) | *WHO alcohol availability [Recorded alcohol per capita consumption (15+ years)]*: Annual volume in liters of pure alcohol per person aged ≥15 available for direct human consumption collected from different sources (World Drink Trends for 2001, availability and taxation for 2002-2010). Categories: alcohol, beer, wine, spirits, and other beverages | 2011 data were imputed from AEAT.  Ref.: <http://apps.who.int/gho/data/node.main.A1026?lang=en> |
| Food and Agriculture Organization (FAO) | *FAO alcohol availability [Food supply quantity of alcoholic beverages]:* Annual supplies in tons of alcoholic beverages available for human food use at retail level within Spain, extracted from supply balance sheets. Categories: beer, wine, other fermented beverages, and other alcoholic beverages. It is calculated as beverage produced plus imported plus stock variation minus that exported, processed, dedicated to nonfood uses, and lost during storage and transportation. | “Wine” includes wine and aperitifs, but it is impossible to disaggregate the two beverages. “Other fermented beverages” includes cider. Mass units were converted into volume by dividing them by beverage density: beer and cider (1.01), wine (0.99), and spirits (0.95).  Ref.: Alcohol supplies: <http://faostat3.fao.org/download/FB/CC/E>  Ref.: Beverage density:<http://www.fao.org/infoods/infoods/tables-and-databases/faoinfoods-databases/en/> |
| Spanish Food Consumption Panel (PCA) | *Self-reported alcohol purchases [Direct demand for alcoholic beverages]*: Annual volumes in millions of liters of alcoholic beverages purchased by households in any retail outlet for consumption elsewhere (off-premise purchases) or by catering establishments or institutions with catering for consumption within the venue (on-premises purchases). Categories: wine, beer, cider, and other. Off- and on-premises purchases are self-recorded daily (scanning barcodes with an optical device) and monthly, respectively, by panelists. Non-alcoholic beer and wine-based alcoholic mixtures (wine mixed with non-alcoholic beverages such as "sangría") were collected separately from 2004 and 2008, respectively^b^. | Annual sample size: 6000-12000 households, 700-1500 catering establishments, and 200-402 institutions.  2011 on-premises purchases were those reported by the PCA to AEAT.  Ref.: <http://www.magrama.gob.es/es/estadistica/temas/estadisticas-alimentacion/consumo-alimentario/> |
| Spanish Tourism Institute (IET)  WHO, Eurostat, World Bank,  Other | *Parameters to estimate consumption and purchases by foreign visitors in Spain and Spanish visitors abroad*: Arrivals and departures of tourists and same-day visitors, tourists’ length of stay, alcohol per capita consumption, comparative index prices of alcohol by country, and price elasticity of alcohol demand. | International visitors: Ref.: IET^c^. <http://estadisticas.tourspain.es/es-ES/turismobase/Paginas/default.aspx>  Alcohol per capita consumption: Ref.: WHO-GISAH. <http://apps.who.int/gho/data/node.main.GISAH?lang=en>  Alcohol price (EU countries): Ref.: Eurostat: <http://ec.europa.eu/eurostat/web/purchasing-power-parities/data/database>  Alcohol price (non-UE countries). Ref.: World Bank: <http://icp.worldbank.org/icp/SelectElements.aspx?r=-1&ds=0&y=1&ws=3>  Price elasticity: Ref.: RAND Corporation: <http://www.rand.org/content/dam/rand/pubs/technical_reports/2009/RAND_TR689.pdf>. |
| National Statistics Institute (INE) | *Official mid-year estimates of population aged ≥15* | Ref.: <http://www.ine.es/jaxiT3/Tabla.htm?t=9663&L=0> |

***[]*** The name in brackets is that assigned by the source to the indicator.

**ABV:** Alcohol-by-volume. It refers to the proportion of the total volume of a liquid that is pure alcohol and is usually expressed as a percent.

**^a^** Direct human consumption refers to the intake as food or for pleasure or other reasons. Intermediate products refer to beverages 1.2%-22% ABV which do not fall under the beer, wine and fermented beverage categories, such as vermouth, sherry or port.

**^b^** Catering establishments include bars, cafes, hotels, hostels, restaurants, etc. Institutions with catering include staff canteens, nursing homes, schools, colleges, hospitals, military establishments, or prisons.

**^c^** To estimate the number of same-day visits of Spanish residents abroad, the French and Portuguese statistics on foreign same-day visitor arrivals 2005-2011 were also used. Ref.: Direction Générale des Entreprises. Etudes et statistiques. Memento du Tourisme. <http://www.entreprises.gouv.fr/etudes-et-statistiques/statistiques-du-tourisme/donnees-cles/memento-du-tourisme>. Ref.:Turismo de Portugal. Anuário das estatísticas do turismo. <http://www.turismodeportugal.pt/Portugu%C3%AAs/ProTurismo/estat%C3%ADsticas/an%C3%A1lisesestat%C3%ADsticas/oturismoem/Pages/OTurismoem.aspx>
