# Supplementary material for: Estimating average alcohol consumption in the population using multiple sources: the case of Spain
Source: Popul Health Metr. 2016 Jun 2;14:21. doi: 10.1186/s12963-016-0090-4 (PMC4890273; doi:10.1186/s12963-016-0090-4)
Supplement: Additional file 2: — Methodological characteristics of main population surveys providing data to calculate self-reported alcohol consumption among population aged ≥15, and calculation algorithms, Spain, 2001–2011. The characteristics considered are: year of the survey, effective sample size, mode of questionnaire administration, questions on frequency of consumption, questions on number of standard drinks, response rate, and percent of missing values for quantity-frequency questions. (DOCX 15 kb) [file 12963_2016_90_MOESM2_ESM.docx]

Additional file 2. Methodological characteristics of main population surveys providing data to calculate self-reported alcohol consumption among population aged ≥15, and calculation algorithms, Spain, 2001-2011

|  | **Spanish National Health Survey** | **Spanish National Health Survey** | **Spanish National Health Survey** | **European Health Survey in Spain^a^** |
| --- | --- | --- | --- | --- |
| **Year** | 2001 | 2006 | 2011 | 2009 |
| **Effective sample size** | 20293 | 28596 | 20776 | 21223 |
| **Questionnaire administration** | PAPI | PAPI | CAPI and exceptionally CATI | CAPI and exceptionally CATI |
| **Categories of alcoholic beverages** | 6: beer, wine, aperitifs, cider, cocktails-brandy-spirits, whisky | 6: beer, wine, aperitifs, cider, cocktails-brandy-spirits, whisky | 6: beer, wine, aperitifs, fruity liqueurs, spirits-cocktails, local beverages | 6: beer, wine, aperitifs, liqueurs, spirits-cocktails, local beverages |
| **Questions on consumption frequency** | One nine-category question for each beverage: nº of drinking episodes a day, week or month (6 questions) | One four-category question on consumption periodicity (daily, weekly, monthly, annually) and one open question on nº of drinking episodes in the selected period for each beverage (12 questions) | One five-category question for alcoholic beverages in general on number of drinking days in last 12 months | One five-category question for alcoholic beverages in general on number of drinking days in last 12 months |
| **Questions on nº of standard drinks** | One open question for each beverage: Nº of standard drinks per drinking episode (6 questions) | One open question for each beverage: Nº of standard drinks per drinking episode (6 questions) | One open question for each beverage and day of the week in a week with normal activity (42 questions) | One open question for each beverage and day of the week in a week with normal activity (42 questions) |
| **Response rate (%)** | _ | 71.6 | 73.3 | 71.1 |
| **Missing values for quantity-frequency questions (%)** | 3.5 | 3.3 | 4.3 | 1.1 |

**^a^**: QF questions are common with other European countries, which would facilitate between-country comparison.

**CAPI**: Computer Assisted Personal Interview. **CATI**: Computer Assisted Telephone Interview. **PAPI**: Paper-and-Pen Interview.

**Effective sample size**: It refers to nº of respondents in which an estimate of alcohol consumption could be obtained.

**Questions on nº of standard drinks**: Standard drinks were named differently in the questionnaire depending on the type of beverage; the name usually referring to the container used for consumption (glasses, cans, bottles, cups, etc.).

**Common characteristics for all surveys**: 1) Reference population: People aged ≥15 residing in family households in Spain. 2) Sampling procedure: Three-stage stratified cluster design. The three stages were census tract, household and individual. First-stage units were stratified by region and municipality size. 3) The reference period for quantity-frequency questions was last 12 months.

**Calculation algorithms of self-reported alcohol consumption**: Self-reported alcohol consumption (*C_SR_*) was expressed in liters of pure alcohol per person-year (lpa/py) and was obtained as *C_SR_=*$\sum_{i=1}^{k} \frac{{(F}_{i}{SD}_{i}A_{i} 0.79)/1000)}{n},$ where subscript *_i_* indicates beverages categories (*k=6*), *F_i_* the annual nº of days or times each beverage is consumed, *SD_i_* the daily nº of standard drinks of each beverage, Ai the volume of pure alcohol in the standard drink of a specific beverage in grams, and 0.79 the alcohol density. To calculate *F_i_* arithmetic midpoints of frequency categories were used (i.e., if frequency of consumption was 4-6 times/week *Fi*=5 x 52.18=260.9 times). A_i_ was directly assigned or calculated as *A_i_=V_i_ C_i_*, where *V_i_* is the assumed volume in liters (l) for the standard drink of each beverage: beer and cider (0.250 l), wine (0.125 l), intermediate products (0.100 l) and spirits and spirit-cocktails (0.060 l), considering in the latter the volume of spirits. C_i_: alcohol content expressed as proportion of alcohol-by-volume in each beverage: beer and cider (0.05), wine (0.115), intermediate products or aperitifs (0.15) and spirits (0.35). Times is a term very often used in questions on frequency of alcohol drinking (times per day, week, month or year). It usually refers to the number of drinking episodes, leaving the interpretation of what is a "drinking episode" at the discretion of the respondent.

**Access to individualized databases and metadata:** Ref.: Instituto Nacional de Estadística. Encuesta Nacional de Salud y Encuesta Europea de Salud (<http://www.ine.es/inebmenu/mnu_salud.htm>) and Ministerio de Sanidad, Servicios Sociales e Igualdad (<http://www.msssi.gob.es/estadEstudios/estadisticas/encuestaNacional/home.htm>)
