# Supplementary material for: Estimating average alcohol consumption in the population using multiple sources: the case of Spain
Source: Popul Health Metr. 2016 Jun 2;14:21. doi: 10.1186/s12963-016-0090-4 (PMC4890273; doi:10.1186/s12963-016-0090-4)
Supplement: Additional file 3: — Algorithms and assumptions to estimate actual per capita alcohol consumption from multisource availability, Spain, 2001–2011. Definition, algorithms, and assumptions are included for the following indicators: actual per capita alcohol consumption, alcohol consumption abroad by Spanish visitors, alcohol imported by Spanish visitors abroad, other unrecorded alcohol, alcohol losses after sale, alcohol consumption in Spain by foreign visitors, and alcohol exported from Spain by foreign visitors. (DOCX 21 kb) [file 12963_2016_90_MOESM3_ESM.docx]

Additional file 3. Algorithms and assumptions to estimate actual per capita alcohol consumption from multisource availability, Spain, 2001-2011

| **Indicator** | **Algorithm** | **Legend, definitions, and assumptions** |
| --- | --- | --- |
| Actual per capita alcohol consumption (*C_P_*) | *C_P_=R+C_SV+_I_SV_+U-L-C_FV_-E_FV_* | ***C_P_*:** Alcohol consumed by residents in Spain aged ≥15. ***R***: Multisource alcohol availability. ***C_SV_*:** Alcohol consumed abroad by Spanish visitors. ***I_SV_*:** Alcohol personally imported from abroad by Spanish visitors. ***U*:** Unrecorded alcohol other than *C_SV_* and *I_SV_*. It includes the alcohol consumed by Spanish residents but unrecorded in routine statistics, including alcohol that is smuggled, surrogate or informally produced, and alcohol from products with ≤1.2% ABV. It does not include alcohol consumed/purchased by Spanish visitors abroad (*C_SV_* and *I_SV_*). ***L***: Alcohol losses after sale. It includes the alcohol spilled, spoiled, wasted, e.g., unfinished drinks, or used for cooking or purposes other than direct human consumption. ***C_FV_*:** Alcohol consumed in Spain by foreign visitors. ***E_FV_*:** Alcohol personally exported from Spain by foreign visitors. All components entered in the algorithm are volumes expressed in liters of pure alcohol per person-year (lpa/py). |
| Alcohol consumption abroad by Spanish visitors (*C_SV_*) | *C_SV=_* $\frac{[\sum_{i=1}^{k} {DS}_{i} AS H \left( \frac{(PS -{PF}_{i}) Ed}{{PF}_{i}}+1 \right)]}{365.25}/N$  ${DS}_{i}=\sum_{i=1}^{k} {ST}_{i} {LS}_{i}+{0.5SE}_{i}$ | $\boldsymbol{DS}_{\boldsymbol{i}}\boldsymbol{:}$ Number of days of stay of Spanish residents in a given foreign country.  ***AS*:** Per capita alcohol consumption in Spain in lpa/py. ***H*:** Holiday factor indicating how many times higher alcohol consumption is in holiday than non-holiday time in a country with the same price index as Spain. As data from elsewhere suggest *H*>1^1^, it was assumed: 1.0≤*H*≤1.5. ***PS*:** Alcohol price index in Spain. ***Ed***: Price elasticity of demand. From international meta-analysis it was assumed 0.40≤*Ed****≤***0.60. ***PF_i_*:** Alcohol price index in a given foreign country (UE27=100). It expresses the price level of alcoholic beverages in a country relative to the average price level in all 27 member states of the European Union (EU-27). For EU countries *PF_i_* was taken directly from Eurostat, while for non-EU countries it was estimated from the comparative price index for alcoholic beverages and tobacco included in the 2005 and 2011 International Comparison Program of the World Bank. *N*: Population resident in Spain aged ≥ 15 at mid-calendar year. $\boldsymbol{ST}_{\boldsymbol{i}}$**:** Nº of tourist visits of Spanish residents to a given foreign country. Tourist visits are those with at least one overnight stay, also including visits for reasons other than entertainment or recreation. $\boldsymbol{LS}_{\boldsymbol{i}}$: Average length of stay of tourist visits of Spanish residents to a given foreign country in days (overnights). ***0.5***: Factor to assume a half-day stay for each same-day visit. $\boldsymbol{SE}_{\boldsymbol{i}}$: Nº of same-day visits of Spanish residents to a given foreign country. Same-day visits (excursionist visits) are those with no overnight stay. Data for France and Portugal in 2005-2011 were directly taken from tourism statistics in those countries (same-day visitors arrivals from Spain), while in 2001-2004 they were obtained by linear projection of 2005-2011 data. Data for the rest of the world were obtained assuming that % of total Spanish same-day visitors abroad who visit France and Portugal was the same as the known % of total foreign same-day visitors to Spain coming from France and Portugal (83.4% in 2011). **Subscript _i_**: Country categories. For ${ST}_{i} \mathrm{and} {LS}_{i}$*k*=10. For ${SE}_{i}$ *k*=3 (France, Portugal and rest of the world). |
| Alcohol imported by Spanish visitors abroad (*I_SV_*) | *I_SV_=*$[\sum_{i=1}^{k} {(ST}_{i}{+SE}_{i})S\left( \frac{AS}{{AF}_{i}} \right) \left( \frac{(PS -{PF}_{i}) Ed}{{PF}_{i}}+1 \right)]/N$ | ***S*:** Souvenir factor indicating the average alcohol purchased abroad and brought to Spain by Spanish residents visiting foreign countries with per capita alcohol consumption and alcohol price index equal to Spain (neutral visitors). Considering European data^2^, values between 0.1 and 0.3 lpa were assumed (0.1 lpa ≈ alcohol content in a 75 cl wine bottle).  ***AF_i_*:** Total alcohol per capita (15+ years) consumption in a given foreign country in lpa/py. Data obtained by linear interpolation of averages 2003-2005, 2008-2010 and 2015 included Global Information System on Alcohol and Health (GISAH)^3^ |
| Other unrecorded alcohol (*U*) | *U=U_T_-C_SV_-I_SV_* | Published estimates for total unrecorded alcohol (*U_T_*) in Spain vary between 1.0 (1997)^2^, 1.4 (2005) and 1.2 lpa/py (2010)^3^. Thus, *U_T_* values of 6%-12% of *R* were assumed. |
| Alcohol losses after sale (*L*) | *L=Lp (R+C_SV_+I_SV_+U)* | Based on some empirical data in other countries suggesting a proportion of alcohol losses (*L_P_*) <10%^4^, in this work values between 6% and 10% of (*R+C_SV_+I_SV_+U*) were assumed. |
| Alcohol consumption in Spain by foreign visitors (*C_FV_* ) | *C_FV =_* $\frac{[\sum_{i=1}^{k} {DF}_{i} {AF}_{i} H \left( \frac{(PS -{PF}_{i}) Ed}{{PF}_{i}}+1 \right) ]}{365.25}/N$  ${DF}_{i}=\sum_{i=1}^{k} {FT}_{i} {LF}_{i}+{0.5FE}_{i}$ | $\boldsymbol{DF}_{\boldsymbol{i}}\boldsymbol{:}$ Total days of stay in Spain of foreign tourists from a given country. ***FT_i_*:** Nº of tourist visits to Spain by residents from a given foreign country. ***LF_i_*:** Average length of stay of tourist visits to Spain by residents from a given foreign country in days (overnight stays). ***FE_i_*:** Nº of same-day visits to Spain by residents from a given foreign country.  **Subscript _i_**: Country categories. For ${FT}_{i} \mathrm{and} {LF}_{i}$*k*=28, for ${FE}_{i}$ *k*=3 (France, Portugal and rest of the world). |
| Alcohol exported from Spain by foreign visitors (*E_FV_* ) | *E_FV_=*$[\sum_{i=1}^{k} {(FT}_{i}+{FE}_{i})S\left( \frac{{AF}_{i}}{AS} \right)\left( \frac{(PS -{PF}_{i}) Ed}{{PF}_{i}}+1 \right)]/N$ | ***S***: Souvenir factor indicating the average alcohol purchases in Spain and taken to the home country for a visitor from a foreign country with per capita alcohol consumption and alcohol price index equal to Spain (neutral visitor). |

.$\left( \frac{(PS -{PF}_{i}) Ed}{{PF}_{i}}+1 \right)$: It includes the effect of between-country price differences in alcohol consumption/purchases by international visitors. It was derived from: [($\frac{Ps}{PFi}-1)Ed]+1$.

^1^: Boniface S., Shelton N. How is alcohol consumption affected if we account for under-reporting? A hypothetical scenario. *Eur J Public Health* 2013; 23: 1076-1081. Trolldal B. Alcohol sales figures in 15 European countries: corrected for consumption abroad and tax-free purchases. *Nordic Studies on Alcohol* 2001; 18: 71-81. ^2^: Norstrom T (Ed). *Alcohol in postwar Europe: Consumption, drinking patterns, consequences and policy responses in 15 European countries*. Stockholm: European Commission; 2001. [http://btg.ias.org.uk/alcohol-policy-eu.html. Accessed 11 Dec 2015](http://btg.ias.org.uk/alcohol-policy-eu.html.%20Accessed%2011%20Dec%202015). ^3^: World Health Organization. Global Health Observatory data repository. Levels of Consumption. <http://apps.who.int/gho/data/node.main.GISAH?showonly=GISAH>. Accessed 11 Dec 2015. ^4^: Boniface S., Shelton N. How is alcohol consumption affected if we account for under-reporting? A hypothetical scenario. *Eur J Public Health* 2013; 23: 1076-1081. Robinson M., Thorpe R., Beeston C., McCartney G. A review of the validity and reliability of alcohol retail sales data for monitoring population levels of alcohol consumption: a Scottish perspective. *Alcohol Alcohol* 2013; 48: 231-240
