# Supplementary material for: Estimating average alcohol consumption in the population using multiple sources: the case of Spain
Source: Popul Health Metr. 2016 Jun 2;14:21. doi: 10.1186/s12963-016-0090-4 (PMC4890273; doi:10.1186/s12963-016-0090-4)
Supplement: Additional file 4: — Results for the different indicators needed to estimate alcohol actual per capita alcohol consumption from multisource alcohol availability under various assumptions, 2001–2011. Liters of pure alcohol/person-year. The results for the main indicators needed to obtain actual per capita alcohol consumption from multisource alcohol availability in the period 2001–2011 are shown. Indicators considered are: alcohol consumed/purchased by Spanish visitors abroad, unrecorded alcohol, alcohol lost, and alcohol consumed/purchased by foreign visitors in Spain. Estimates were obtained under intermediate, high, and low assumptions. (DOCX 18 kb) [file 12963_2016_90_MOESM4_ESM.docx]

Additional file 4. Results for the different indicators needed to estimate alcohol actual per capita alcohol consumption from multisource alcohol availability under various assumptions, 2001-2011. Liters of pure alcohol/person-year.

|  |  | **Alcohol components** | | | | | | | | | | | | |
| --- | --- | --- | --- | --- | --- | --- | --- | --- | --- | --- | --- | --- | --- | --- |
|  |  | Multisource availability (*R*) |  | Consumed/purchased by Spanish visitors abroad | |  | Unrecorded (*U*) |  | Lost (*L*) |  | Consumed/purchased by foreign visitors in Spain | |  | Per capita consumption (*C_P_*) |
| **Year** |  |  |  | Consumed (*C_FV_*) | Purchased (*I_FV_*) |  |  |  |  |  | Consumed (*C_FV_*) | Purchased (*E_FV_*) |  |  |
| **Intermediate estimate** | | | | | | | | | | | | | | |
| 2001 |  | 14.0 |  | 0.1 | 0.1 |  | 1.0 |  | 1.2 |  | 0.9 | 0.6 |  | 12.5 |
| 2002 |  | 12.3 |  | 0.1 | 0.1 |  | 0.9 |  | 1.1 |  | 0.8 | 0.6 |  | 10.9 |
| 2003 |  | 13.0 |  | 0.1 | 0.1 |  | 0.9 |  | 1.1 |  | 0.8 | 0.5 |  | 11.7 |
| 2004 |  | 13.3 |  | 0.1 | 0.2 |  | 0.9 |  | 1.2 |  | 0.8 | 0.6 |  | 12.0 |
| 2005 |  | 12.7 |  | 0.1 | 0.2 |  | 0.8 |  | 1.1 |  | 0.7 | 0.6 |  | 11.4 |
| 2006 |  | 12.8 |  | 0.1 | 0.2 |  | 0.8 |  | 1.1 |  | 0.7 | 0.6 |  | 11.5 |
| 2007 |  | 12.5 |  | 0.1 | 0.2 |  | 0.8 |  | 1.1 |  | 0.7 | 0.6 |  | 11.2 |
| 2008 |  | 11.6 |  | 0.1 | 0.2 |  | 0.7 |  | 1.0 |  | 0.6 | 0.5 |  | 10.4 |
| 2009 |  | 11.1 |  | 0.1 | 0.2 |  | 0.6 |  | 1.0 |  | 0.6 | 0.5 |  | 10.0 |
| 2010 |  | 10.8 |  | 0.1 | 0.2 |  | 0.6 |  | 0.9 |  | 0.5 | 0.5 |  | 9.7 |
| 2011 |  | 10.6 |  | 0.1 | 0.2 |  | 0.5 |  | 0.9 |  | 0.6 | 0.5 |  | 9.5 |
| 2001-2011 |  | 12.2 |  | 0.1 | 0.2 |  | 0.8 |  | 1.1 |  | 0.7 | 0.6 |  | 10.9 |
| **Low estimate** | | | | | | | | | | | | | | |
| 2001 |  | 14.0 |  | 0.1 | 0.1 |  | 0.7 |  | 1.5 |  | 1.1 | 0.9 |  | 11.3 |
| 2011 |  | 10.6 |  | 0.1 | 0.1 |  | 0.4 |  | 1.1 |  | 0.7 | 0.8 |  | 8.6 |
| 2001-2011 |  | 12.2 |  | 0.1 | 0.1 |  | 0.5 |  | 1.3 |  | 0.9 | 0.9 |  | 9.9 |
| **High estimate** | | | | | | | | | | | | | | |
| 2001 |  | 14.0 |  | 0.1 | 0.2 |  | 1.4 |  | 0.9 |  | 0.7 | 0.3 |  | 13.8 |
| 2011 |  | 10.6 |  | 0.2 | 0.3 |  | 0.7 |  | 0.7 |  | 0.4 | 0.3 |  | 10.4 |
| 2001-2011 |  | 12.2 |  | 0.1 | 0.3 |  | 1.0 |  | 0.8 |  | 0.5 | 0.3 |  | 12.0 |

**Liters of pure alcohol/person-year:** It was calculated by dividing the total volume of pure alcohol in liters of each component in the algorithm by official estimates of mid-year population aged ≥15.

***R***: Multisource alcohol availability. Obtained from sales recorded by Spanish Tax Agency by replacing wine figures by Eurostat wine retail supplies, and adding FAO cider retail supplies; ***C_SV_*:** Alcohol consumed abroad by Spanish visitors; ***I_SV_*:** Alcohol purchased abroad by Spanish visitors and imported to Spain; ***U***: Unrecorded alcohol other than *C_SV_* and *I_SV_*; ***L***: Alcohol lost after sale, ***C_FV_***: Alcohol consumed in Spain by foreign visitors, ***E_FV_***: Alcohol purchased in Spain by foreign visitors and personally exported from Spain, ***C_P_***: Alcohol consumed by population resident in Spain aged ≥15, *C_P_=R+C_SV+_I_SV_+U-L-C_FV_-E_FV_*; *U=U_T_-C_SV_-I_SV_*, where *U_T_* is total unrecorded alcohol. See calculation algorithms of the different parameters in Appendix 3.

**Intermediate estimate**: Assumed parameter values: Souvenir factor (*S*) for foreign visitors to Spain and Spanish visitors abroad= 0.2 lpa/visitor; Holiday factor (*H*) for foreign visitors to Spain and Spanish visitors abroad=1.25, *U_T_*=9% of *R*, with a relative decline of 1% annually, *L*=8% of (*R+C_SV_+I_SV_+U*).

**Low estimate**: Assumed parameter values: *S* for foreign visitors to Spain=0.3 lpa/visitor, *S* for Spanish visitors abroad= 0.1 lpa/visitor; *H* for foreign visitors to Spain=1.5, *H* for Spanish visitors abroad=1.0, *U_T_*=6% of *R* with a relative decline of 1% annually, *L*=10% of (*R+C_SV_+I_SV_+U*).

**High estimate:** Assumed parameter values: *S* for foreign visitors to Spain=0.1 lpa/visitor, *S* for Spanish visitors abroad= 0.3 lpa/visitor; *H* for foreign visitors to Spain=1.0, *H* for Spanish visitors abroad=1.5, *U_T_* =12% of *R*, with a relative decline of 1% annually, *L*=6% of (*R+C_SV_+I_SV_+U*).
