# Supplementary material for: Estimating average alcohol consumption in the population using multiple sources: the case of Spain
Source: Popul Health Metr. 2016 Jun 2;14:21. doi: 10.1186/s12963-016-0090-4 (PMC4890273; doi:10.1186/s12963-016-0090-4)
Supplement: Additional file 5: — Example of estimation of alcohol consumed/purchased in visits to Spain by foreign residents and visits abroad by Spanish residents, 2011. Data for different countries on parameters needed for the estimation are included, specifically on: total alcohol per capita consumption, alcohol price index, number of inbound and outbound visits, and number of inbound and outbound stays. The calculations are disaggregated for overnight visitors and same-day visitors. (DOCX 24 kb) [file 12963_2016_90_MOESM5_ESM.docx]

Additional file 5. Example of estimation of alcohol consumed/purchased in visits to Spain by foreign residents and visits abroad by Spanish residents, 2011

|  | | **Total alcohol per capita (*AFi)*** | | |  | **Alcohol price (PFi)** | |  | **Visits (*FV_i_*)** | | **Stays (*DF_i_*)** | | **Alcohol consumed or exported in the visit** | | | | | | | | | |  | | **Visits (*SV_i_*)** | | **Stays (*DS_i_*)** | | **Alcohol consumed or imported in the visit** | | | | | | | |  |
| --- | --- | --- | --- | --- | --- | --- | --- | --- | --- | --- | --- | --- | --- | --- | --- | --- | --- | --- | --- | --- | --- | --- | --- | --- | --- | --- | --- | --- | --- | --- | --- | --- | --- | --- | --- | --- | --- |
|  | |  | | |  |  | |  |  | |  | | ***C_FV_*** | | | ***E_FV_*** | | | ***T_FV_*** | | | |  | |  | |  | | ***C_SV_*** | | | ***I_SV_*** | | | ***T_SV_*** | |  |
|  | |  | | |  |  | |  | **Overnight foreign visitors to Spain** | | | | | | | | | | | | | |  | | **Overnight Spanish visitors abroad** | | | | | | | | | | | |  |
| Germany | | 11.6 | | |  | 83.7 | |  | 8975 | | 89752 | | | 0.09 | | | 0.04 | | | 0.13 | | | |  | | 766 | 5494 | | | 0.01 | | | 0.00 | | | 0.01 | |
| Austria | | 9.9 | | |  | 96.1 | |  | 579 | | 5215 | | | 0.00 | | | 0.00 | | | 0.01 | | | |  | |  |  | | |  | | |  | | |  | |
| Belgium | | 11.0 | | |  | 98.5 | |  | 1757 | | 17567 | | | 0.02 | | | 0.01 | | | 0.03 | | | |  | |  |  | | |  | | |  | | |  | |
| Denmark | | 11.2 | | |  | 136.1 | |  | 910 | | 9095 | | | 0.01 | | | 0.01 | | | 0.02 | | | |  | |  |  | | |  | | |  | | |  | |
| Finland | | 12.2 | | |  | 169.5 | |  | 574 | | 5740 | | | 0.01 | | | 0.00 | | | 0.01 | | | |  | |  |  | | |  | | |  | | |  | |
| France | | 12.1 | | |  | 89.3 | |  | 8375 | | 67000 | | | 0.07 | | | 0.04 | | | 0.12 | | | |  | | 2423 | 13832 | | | 0.01 | | | 0.01 | | | 0.02 | |
| Ireland | | 11.7 | | |  | 169.6 | |  | 1284 | | 11558 | | | 0.02 | | | 0.01 | | | 0.03 | | | |  | |  |  | | |  | | |  | | |  | |
| Italy | | 6.6 | | |  | 99.2 | |  | 3765 | | 30119 | | | 0.02 | | | 0.01 | | | 0.03 | | | |  | | 1516 | 10406 | | | 0.01 | | | 0.01 | | | 0.02 | |
| Norway | | 7.6 | | |  | 279.7 | |  | 1120 | | 11203 | | | 0.02 | | | 0.01 | | | 0.02 | | | |  | |  |  | | |  | | |  | | |  | |
| Netherlands | | 9.8 | | |  | 98.1 | |  | 2772 | | 27719 | | | 0.03 | | | 0.01 | | | 0.04 | | | |  | |  |  | | |  | | |  | | |  | |
| Portugal | | 12.8 | | |  | 89.1 | |  | 1878 | | 9390 | | | 0.01 | | | 0.01 | | | 0.02 | | | |  | | 1634 | 8330 | | | 0.01 | | | 0.01 | | | 0.02 | |
| UK | | 11.7 | | |  | 134.9 | |  | 13615 | | 122538 | | | 0.16 | | | 0.09 | | | 0.25 | | | |  | | 1146 | 9502 | | | 0.01 | | | 0.00 | | | 0.01 | |
| Russia | | 15.0 | | |  | 44.1 | |  | 863 | | 7766 | | | 0.01 | | | 0.00 | | | 0.01 | | | |  | |  |  | | |  | | |  | | |  | |
| Sweden | | 9.1 | | |  | 160.6 | |  | 1294 | | 12942 | | | 0.01 | | | 0.01 | | | 0.02 | | | |  | |  |  | | |  | | |  | | |  | |
| Switzerland | | 10.6 | | |  | 122.0 | |  | 1367 | | 12300 | | | 0.01 | | | 0.01 | | | 0.02 | | | |  | |  |  | | |  | | |  | | |  | |
| Rest of Europe^a^ | | 10.8 | | |  | 110.7 | |  | 2292 | | 20625 | | | 0.02 | | | 0.01 | | | 0.03 | | | |  | | 2974 | 21493 | | | 0.02 | | | 0.01 | | | 0.03 | |
| USA | | 9.2 | | |  | 106.7 | |  | 1137 | | 15922 | | | 0.01 | | | 0.01 | | | 0.02 | | | |  | |  |  | | |  | | |  | | |  | |
| Argentina | | 9.0 | | |  | 70.8 | |  | 307 | | 3988 | | | 0.00 | | | 0.00 | | | 0.00 | | | |  | |  |  | | |  | | |  | | |  | |
| Brazil | | 8.8 | | |  | 75.3 | |  | 370 | | 4809 | | | 0.00 | | | 0.00 | | | 0.00 | | | |  | |  |  | | |  | | |  | | |  | |
| Canada | | 10.2 | | |  | 160.7 | |  | 216 | | 2809 | | | 0.00 | | | 0.00 | | | 0.00 | | | |  | |  |  | | |  | | |  | | |  | |
| Chile | | 9.5 | | |  | 80.0 | |  | 38 | | 490 | | | 0.00 | | | 0.00 | | | 0.00 | | | |  | |  |  | | |  | | |  | | |  | |
| Mexico | | 7.1 | | |  | 67.3 | |  | 259 | | 3367 | | | 0.00 | | | 0.00 | | | 0.00 | | | |  | |  |  | | |  | | |  | | |  | |
| Venezuela | | 8.8 | | |  | 121.3 | |  | 109 | | 1422 | | | 0.00 | | | 0.00 | | | 0.00 | | | |  | |  |  | | |  | | |  | | |  | |
| Rest of America^a^ | | 8.4 | | |  | 88.5 | |  | 451 | | 5859 | | | 0.00 | | | 0.00 | | | 0.01 | | | |  | |  |  | | |  | | |  | | |  | |
| America | | 9.2 | | |  | 106.7 | |  |  | |  | | |  | | |  | | |  | | | |  | | 1462 | 26874 | | | 0.02 | | | 0.01 | | | 0.03 | |
| Japan | | 7.3 | | |  | 114.2 | |  | 343 | | 3773 | | | 0.00 | | | 0.00 | | | 0.00 | | | |  | |  |  | | |  | | |  | | |  | |
| Asia | | 3.8 | | |  | 65.8 | |  |  | |  | | |  | | |  | | |  | | | |  | | 407 | 5449 | | | 0.01 | | | 0.01 | | | 0.01 | |
| Africa | | 6.0 | | |  | 61.0 | |  |  | |  | | |  | | |  | | |  | | | |  | | 1011 | 11054 | | | 0.01 | | | 0.01 | | | 0.03 | |
| Rest of world^a^ | | 6.2 | | |  | 85.1 | |  | 1527 | | 16798 | | | 0.01 | | | 0.00 | | | 0.01 | | | |  | | 8 | 156 | | | 0.00 | | | 0.00 | | | 0.00 | |
| Total | |  | | |  |  | |  | 56177 | | 519766 | | | 0.55 | | | 0.30 | | | 0.85 | | | |  | | 13347 | 112589 | | | 0.11 | | | 0.08 | | | 0.19 | |
| Spain | | 11.6 | | |  | 86.0 | |  |  | |  | | |  | | |  | | |  | | | |  | |  |  | | |  | | |  | | |  | |
|  | |  | | |  |  | |  |  | |  | | |  | | |  | | |  | | | |  | |  |  | | |  | | |  | | |  | |
|  | |  | | |  |  | |  | **Same-day foreign visitors to Spain** | | | | | | | | | | | | | | |  | | **Same-day Spanish visitors abroad** | | | | | | | | | | | |
| France | |  | | |  |  | |  | 24608 | | 12304 | | | 0.01 | | | 0.13 | | | 0.14 | | | |  | | 12549 | 6274 | | | 0.01 | | | 0.06 | | | 0.07 | |
| Portugal | |  | | |  |  | |  | 11277 | | 5639 | | | 0.00 | | | 0.06 | | | 0.06 | | | |  | | 12302 | 6151 | | | 0.01 | | | 0.05 | | | 0.06 | |
| Rest of world^a^ | |  | | |  |  | |  | 7125 | | 3563 | | | 0.00 | | | 0.04 | | | 0.04 | | | |  | | 4117 | 2058 | | | 0.00 | | | 0.01 | | | 0.02 | |
| Total | |  | | |  |  | |  | 43010 | | 21505 | | | 0.02 | | | 0.23 | | | 0.25 | | | |  | | 28968 | 14484 | | | 0.01 | | | 0.13 | | | 0.14 | |
|  | |  | | |  |  | |  | **Total foreign visitors to Spain** | | | | | | | | | | | | | | |  | | **Total Spanish visitors abroad** | | | | | | | | | | | |
| Total | |  | | |  |  | |  | 99187 | | 541271 | | | 0.57 | | | 0.54 | | | 1.10 | | | |  | | 42314 | 127072 | | | 0.12 | | | 0.21 | | | 0.33 | |
|  | |  | |  |  | | |  |  | |  | |  | | |  | | |  | | |  |  | | | | | |  | | |  | | |  |  | |

***AFi***: Total alcohol per capita (15+ years) consumption in a given foreign country in liters of pure alcohol per person-year (lpa/py). ***PFi*:** Alcohol price index in a given foreign country (UE27=100). ***FVi*:** Nº of visits of foreign residents to Spain in thousands. ***DFi*:** Nº of days of stay of foreign visitors in Spain (overnight stays) in thousands. ***C_FV_*:** Alcohol consumed in Spain by foreign visitors in lpa/py. ***E_FV_***: Alcohol personally exported from Spain by foreign visitors in lpa/py. ***T_FV_*:** Total alcohol consumed in Spain or personally exported from Spain by foreign visitors in lpa/py. ***SVi*:** Nº of visits abroad by residents in Spain in thousands. ***DSi*:** Nº of days of stay of Spanish residents in a given country in thousands. ***C_SV_***: Alcohol consumed by Spanish visitors abroad in lpa/py. ***I_SV_***: Alcohol personally imported from abroad by Spanish visitors in lpa/py. ***T_SV_***: Total alcohol consumed abroad or personally imported to Spain by Spanish visitors abroad in lpa/py.

Parameters for all of Europe, America and the world were assigned, respectively, to visits classified as rest of Europe, rest of America and rest of world.

**Overnight international visitors:** International visitors with at least one overnight stay in another country. They are also called international tourists.

**Same-day international visitors**: International visitors with no overnight in another country. They are also called international excursionists.
